# A Synopsis of a Singular Case of Transposition of Viscera, Reported to the Medical Society of Delaware, at Its Annual Session, Held at Dover, June 8th, 1853

**Published:** 1853-11

**Authors:** Henry F. Askew

**Affiliations:** Wilmington, late President


*A Synopsis of a singular case of Transposition of Viscera, reported to the Medical Society of Delaware, at its Annual Session, held at Dover, June 8th,* 1853. By Henry F. Askew, M. D., of Wilmington, late President.

The subject was a little girl, æt. seven years, and daughter of ——— ———, of the city of Wilmington, Delaware. During the month of March, 1853, she suffered for several weeks from an attack of typhoid fever, accompanied with periodical cephalalgia. From these she apparently entirely recovered, and on the 12th of June, she again became indisposed. Her illness was supposed to proceed from eating unripe fruit. Medical attention, however, was not sought until the afternoon of the 16th, and then on account of convulsions, which, though mitigated, were not entirely relieved, except by death. About twelve hours after death the following autopsic phenomena were revealed, viz., the dura mater strongly adherent to the cranium with exudation of lymph, the blood vessels exceedingly injected with dark blood, and spots of lymph beneath the dura mater. The ventricles contained a large amount of serum, and the substance of the brain appeared softened.

A considerable number of cherry stones were found in the alimentary canal; the glands of Peyer had been diseased, and were evidently healing. The mesenteric glands were greatly enlarged. The viscera of the thorax and abdomen were entirely transposed, so that the appearance was such as would be presented from a reflected surface. The apex of the heart was on the right of the median line, the aorta arching off to the right, and extending down the right side of the spinal column. The right lung had but two lobes, the left lung had three. The liver was mostly on the left side of the body, with the gall bladder also on the left side, whilst the spleen occupied its corresponding position on the right. The stomach had its cardiac orifice on the right and the pyloric on the left. The caput colli was attached to the left side, and the descending colon was on the right.

The health of both parents is good, and the size of the little patient was such as generally corresponds to her age. Her health, until the attack of typhoid fever, had always been excellent, and no occurrence had ever led to the suspicion of the transposition which was developed.

